# Fuzzy Coordination Control Strategy and Thermohydraulic Dynamics Modeling of a Natural Gas Heating System for In Situ Soil Thermal Remediation

**DOI:** 10.3390/e21100971

**Published:** 2019-10-05

**Authors:** Zhuang-Zhuang Zhai, Li-Man Yang, Yun-Ze Li, Hai-Feng Jiang, Yuan Ye, Tian-Tian Li, En-Hui Li, Tong Li

**Affiliations:** 1 School of Automation Science and Electrical Engineering, Beihang University, Beijing 100191, China; zhaizz@buaa.edu.cn (Z.-Z.Z.); ylm@buaa.edu.cn (L.-M.Y.); 2 School of Aeronautic Science and Engineering, Beihang University, Beijing 100191, China; litiantian@buaa.edu.cn (T.-T.L.); lienhui@buaa.edu.cn (E.-H.L.); 3 Institute of Engineering Thermophysics, North China University of Water Resources and Electric Power, Zhengzhou 450045, China; 4 Advanced Research Center of Thermal and New Energy Technologies, Xingtai Polytechnic College, Xingtai 054035, China; 5 CENTER International Group Company Limited, Beijing 100176, China; jianghaifeng@centerint.com (H.-F.J.); yeyuan@centerint.com (Y.Y.); 6 Chengyi Academy of PKUHS, Peking University, Beijing 100080, China; litong@i.pkuschool.edu.cn

**Keywords:** soil contamination, in situ thermal remediation, natural gas heating system, fuzzy coordination control strategy, energy consumption

## Abstract

Soil contamination remains a global problem. Among the different kinds of remediation technologies, in situ soil thermal remediation has attracted great attention in the environmental field, representing a potential remedial alternative for contaminated soils. Soils need to be heated to a high temperature in thermal remediation, which requires a large amount of energy. For the natural gas heating system in thermal remediation, a fuzzy coordination control strategy and thermohydraulic dynamics model have been proposed in this paper. In order to demonstrate the superiority of the strategy, the other three traditional control strategies are introduced. Analysis of the temperature rise and energy consumption of soils under different control strategies were conducted. The results showed that the energy consumption of fuzzy coordination control strategy is reduced by 33.9% compared to that of the traditional control strategy I, constant natural gas flow and excess air ratio. Further, compared to the traditional control strategy II, constant excess air ratio and desired outlet temperature of wells, the strategy proposed can reduce energy consumption by 48.7%. The results illustrate the superiority of the fuzzy coordination control strategy, and the strategy can greatly reduce energy consumption, thereby reducing the cost of in situ soil thermal remediation.

## 1. Introduction

Soil pollution is a major global environmental problem [1,2]. For instance, according to the first national pollution survey in China, released in 2014, 16% of the surveyed land (6.3 million km^2^) was contaminated beyond acceptable standards [3]. Meanwhile, 342,000 sites of known contamination and a further 2.5 million potentially polluted sites are present in Europe [4]. Due to the severity of soil pollution and the difficulty of its restoration, the remediation of contaminated soil has attracted great attention in the environmental field.

A cornucopia of remediation technologies has been developed to treat contaminated soils [5,6,7,8]. Among these technologies, in situ soil thermal remediation holds an important niche role due to its ability to clean pollutants quickly and reliably [9]. Thermal remediation is a soil remediation process in which heat and vacuum are applied simultaneously to contaminated soils [10]. After the thermal treatment of soils, the vaporized contaminants will be passed through a pollutant treatment system and released into the atmosphere. Then, the treated soil is available for reuse [11]. Studies have indicated that there is no dispersion of pollutants beyond the boundaries of the treated zone in the course of thermal remediation [12]. Meanwhile, TerraTherm Environmental Services, Inc. (TESI) has made a great effort to detect migration in field experiments and demonstrations, and none of these investigations have found evidence of migration after thermal treatment. Therefore, thermal remediation is very effective for treating contaminated soils, and it does not pose a threat to site workers and the community when properly operated. Thermal remediation has been widely applied to the treatment of organic contaminated sites in American and European countries [13,14,15].

Thermal remediation is mainly applied to the removal of volatile, semivolatile organic pollutants, and a small number of volatile inorganic substances such as Hg, As, and Se [16]. Thermal remediation has many advantages over other remediation methods. Firstly, soil temperature can be increased to higher values through thermal remediation, if desired, to values on the order of 600 °C [17]; thus, it can meet cleanup standards for lots of contaminants [18]. Furthermore, thermal remediation is effective for uniformly heating the entire contamination zone [19]. Because thermal conductivity of soils is much less variable, it changes by only a factor of approximately 4 from clay to sand [20]. Further, in situ thermal remediation can be used deep underground or beneath buildings, which would otherwise be difficult or costly to dig up to treat above ground. Thermal remediation can also be used in silty or clayey soils, where other treatment technologies do not perform well. Compared to ex situ, in situ thermal remediation involves no digging and hauling of contaminated soils, generates no dust or odors, minimizes human exposure to hazardous wastes, and is a low-noise operation [21]. Further, in the first full-scale commercial application of in situ thermal remediation, new grass growth reappeared naturally without reseeding after the remediation operation was completed. No damage was observed to the nearby trees [12,22]. Some of the literature has focused on the changes of soil properties after thermal treatment [11,23], and it was found that treated soils have a high abundance and diversity of microorganisms and fauna, but enzymatic activities are weak. Thus, the treated soils may still be suitable for sustaining vegetation, though likely at a slightly diminished capacity when compared with native soils. On the other hand, in situ thermal remediation may be more ecofriendly in sensitive ecosystems because of the lack of soil disturbance [24].

According to the source of energy in thermal remediation, it can be divided into natural gas heating and electric heating. Natural gas heating is more energy efficient than electric heating, but it is accompanied by problems with the control of natural gas flow. A great deal of effort has gone into developing thermal remediation to remove or reduce contaminants in soils. As soil is heated, contaminants in soils are vaporized or destroyed by a number of mechanisms. Heron et al. [25] presented the largest thermal remediation project and the associated challenges of constructing and treating quickly. In this project, an estimated 13,400 kg of chlorinated volatile organic compounds (CVOC) mass was removed, and all soil goals were met. Chang et al. [26] developed the speciation model for mercury over a range of environmental conditions to identify distribution of dissolved mercury species and potential transformations of mercury during thermal remediation. Falciglia et al. [27] used a laboratory scale apparatus to treat five soil size aggregate fractions, and the effect of soil texture on contaminant adsorption and removal was investigated.

Based on the systematic parametric studies mentioned above, soil temperature was recognized as the key parameter for thermal remediation [28]. Because soils have relatively low heat capacity, the initial heating of the site may require long periods of energy input before contaminants vaporize. During the thermal remediation process, heating contaminated soils to high temperatures is energy-intensive and, thus, a relatively costly endeavor [24,29]. The treatment costs for soils contaminated with semivolatile organic compounds can be high as $550–770 per metric ton [30]. However, in most cases, the studies focus on the chemical modification of contaminants and the removal of pollutants [31,32,33], and the question of how to reduce energy consumption through an appropriate control strategy is ignored. At present, the heating system of thermal remediation is operated under a simple control strategy that leads to a large amount of energy consumed. 

In this paper, a fuzzy coordination control strategy and thermohydraulic dynamics model of natural gas heating systems are proposed for in situ soil thermal remediation. In order to verify the superiority of the strategy, the other three traditional control strategies are introduced. Analysis of the temperature rise and energy consumption of soils under different control strategies was conducted. In Section 2 of this paper, the thermohydraulic models and fuzzy coordination control strategy of heating system are proposed. Section 3 presents the numerical analysis of heating process of soils under four different control strategies. Finally, based on the numerical study results, Section 4 presents the conclusions.

## 2. System Description and Dynamical Modeling

**Figure 1:**
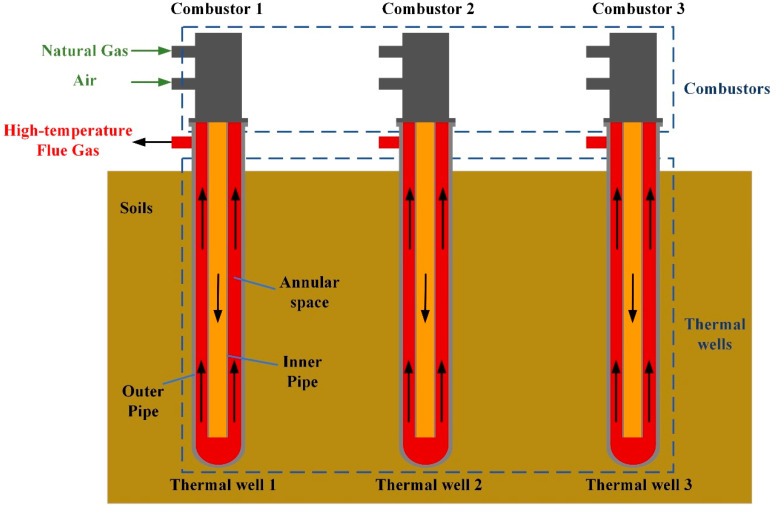
Schematic diagram of a natural gas heating system for in situ soil thermal remediation.

In the process of thermal remediation, the entire treatment zone is heated by an array of natural gas heating devices, as Figure 1 shows. The device is composed of a combustor and a thermal well, and the combustor is fixed on the top of wells. The natural gas and air burn in the combustor. Then, the high-temperature flue gases generated flow into the inner pipe of thermal wells. The gases continue to flow downward to the bottom of thermal wells and enter the annular space. Then, the flue gases flow upward away from thermal wells. In thermal remediation, heat is transferred to the outer wall of thermal well’s outer pipe by convective heat transfer and heat conduction. After that, soil is heated to high temperatures to achieve the removal of pollutants. The objective of this paper is to propose a control strategy to accomplish energy-saving heating of soils by adjusting the natural gas flow and air flow during the heating process.

### 2.1. The Whole Frame of Control Strategy

**Figure 2:**
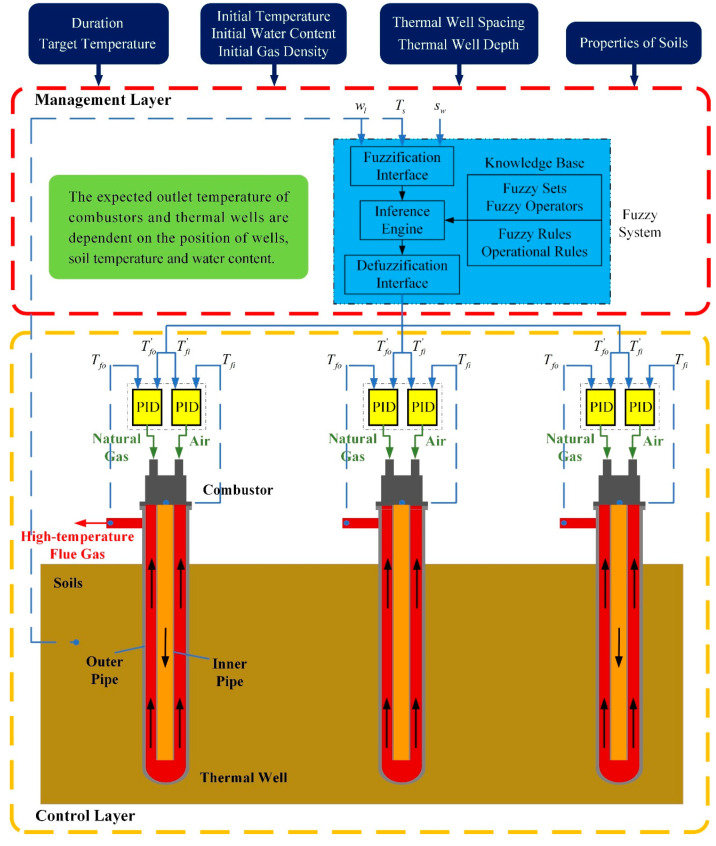
The whole frame of control strategy.

Considering the heat leakage to surrounding unheated zones and the moisture migration during the heating process in soils, the requirements of energy at different locations are different. At the same time, the energy demand will be variable when soil temperature changes. On account of these theories, the fuzzy coordination control strategy is proposed. The schematic of control strategy is shown in Figure 2, consisting of a management layer and a control layer.

The management layer is a fuzzy system, which puts out the expected outlet temperature of combustors $T_{fi}^{\prime }$ and thermal wells $T_{fo}^{\prime }$ in accordance with the position of wells $s_{w}$, soil temperature $T_{s}$, and water content $w_{l}$. In this layer, the fuzzy sets, membership functions of variables, operation rules, and fuzzy rules will be determined. 

The control layer contains multiple heating devices inserted into soils. There are two proportional-integral-derivative (PID) controllers, a combustor and a thermal well in each device. Two PID controllers adjust the natural gas flow and air flow separately. In order to analyze the superiority of control strategy, the mathematical model of heating field, thermal wells, and combustors is developed in this layer.

### 2.2. Thermohydraulic Dynamics Modeling

#### 2.2.1. Model of Heating Field

Soil is a typical porous media containing a solid phase, liquid phase, and gas phase. There are pores among soil particles, which are filled with liquid and gas. Studies have shown that the liquid phase and gas phase in soils will migrate from high temperature zone to low temperature zone under the heat drive, which would cause the redistribution of the soil moisture field. The essence of moisture transfer under non-isothermal conditions is the migration of energy, so moisture transfer also affects the change of soil temperature field. Heat and moisture transfer in soils under the temperature gradient and humidity gradient are mainly analyzed in this study.

**Figure 3:**
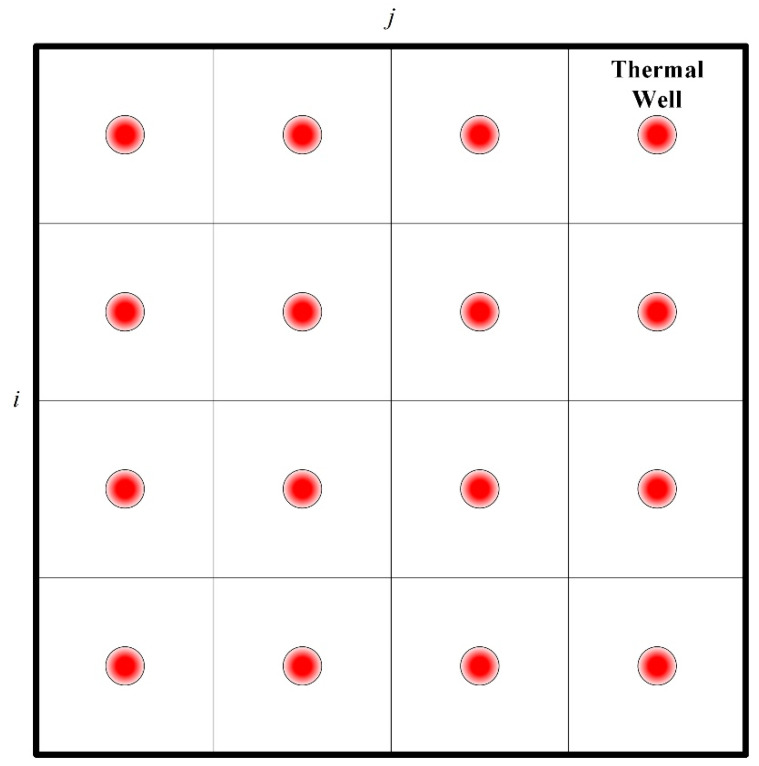
The pattern of thermal wells in treatment zones.

In thermal remediation, soils are heated by an array of thermal wells. Therefore, the whole site is divided into different unit blocks according to the arrangement of thermal wells, as Figure 3 shows. In the lumped parameter method, it is assumed that soil temperature, water content, and other physical parameters at different locations in a certain unit block are the same, and this method puts emphasis on the difference of heating processes in different blocks. (i,j) indicates the number of each unit block.

In order to simulate the heat and moisture transfer in unsaturated soils, the following simplifying assumptions were made in this study.

(1) Soil is homogeneous and its type does not change along thermal wells, while soil is considered, in a real situation, a non-homogeneous and non-isotropic porous material. The effect of this assumption on heat conduction can be negligible because thermal conductivities of different dry soils are much less variable. By contrast, fluid flow permeabilities of different layers may vary much [12], so the permeabilities of soils at different locations are actually a little variable.
(2) Convection of fluid in porous media satisfies Darcy’s law.
(3) The gas phase in soils includes noncondensable gases (such as air) and water vapor. The influence of dry air on heat and moisture migration is neglected.
(4) There is no chemical interaction, and the gas is assumed to be ideal gas.
(5) It is assumed that the solid, liquid, and gas phases are continuous in unsaturated soil, separately.
(6) It is assumed that the migration of liquid and gas do not affect each other.
(7) The compressive work and viscous dissipation effects of liquid are negligible.
(8) The effects of contaminants are ignored.

(a) Liquid Flow Model

**Figure 4:**
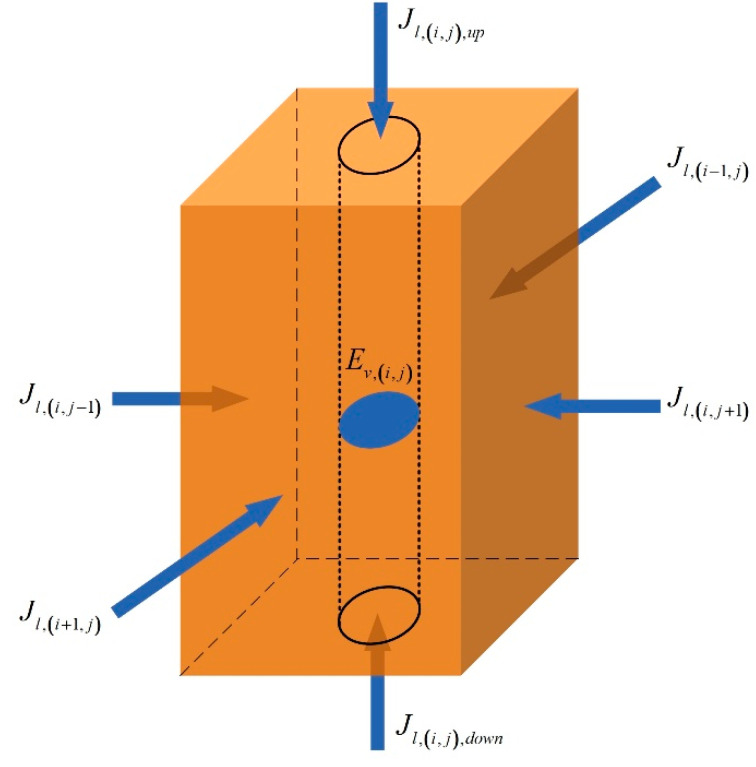
Liquid flow of a unit block.

For the mass conservation, the variable quantity of liquid water in a certain unit block is equal to the difference between the amount of migration from the surrounding units and the amount of inside evaporation. As Figure 4 shows, for a single unit block, the mass balance equation of liquid is:

(1) $$\begin{array}{c} \rho_{l}V_{T,\left(i,j\right)}\frac{dw_{l,\left(i,j\right)}}{d\tau }=E_{l,\left(i,j\right)}+\left(J_{l,\left(i-1,j\right)}+J_{l,\left(i+1,j\right)}+J_{l,\left(i,j-1\right)}+J_{l,\left(i,j+1\right)}+J_{l,\left(i,j\right),down}+J_{l,\left(i,j\right),up}\right)\\ +\left(J_{l,\left(i,j\right),e}+J_{l,\left(i,j\right),w}+J_{l,\left(i,j\right),s}+J_{l,\left(i,j\right),n}\right) \end{array}$$

where $\rho_{l}$ is the density of liquid water. $V_{T,\left(i,j\right)}$ and $w_{l,\left(i,j\right)}$ indicate, respectively, total volume and volume liquid moisture content of the unit (i,j), $V_{T,\left(i,j\right)}=V_{s,\left(i,j\right)}+V_{l,\left(i,j\right)}+V_{v,\left(i,j\right)}$, $w_{l}=\frac{V_{l}}{V_{T}}$. $J_{l}$ is the mass of liquid migrated from adjacent units in unit time. $E_{l,\left(i,j\right)}$ indicates the mass of liquid evaporated in unit time, and it is negative.

According to the Philip and De Vries model [34], the liquid migration equation in unsaturated soils is:

(2) $$j_{l}=-\rho_{l}K_{l}\nabla \psi =-\rho_{l}K_{l}\left(\frac{\partial \psi }{\partial T}\nabla T+\frac{\partial \psi }{\partial w}\nabla w\right)$$

where $j_{l}$ indicates liquid migration mass of unit area in unit time. $K_{l}$ and ψ are hydraulic conductivity and soil water potential, respectively.

The moisture infiltration on the surface of soils is negligible. In thermal remediation, there are vacuum wells to remove gases in soils, and the extraction of liquid is ignored, so $J_{l,\left(i,j\right),up}=0$. In the heating process, there is water infiltration from the unheated zone. These values are set to invariable in this study, as $J_{l,\left(i,j\right),e}$, $J_{l,\left(i,j\right),s}$, $J_{l,\left(i,j\right),n}$, and $J_{l,\left(i,j\right),down}$.

For the lumped parameter method, the liquid fluxes of unit (i,j) with other adjacent units are:

(3) $$\{\begin{array}{l} J_{l,\left(i-1,j\right)}=A_{sd}\rho_{l}\left(D_{lw}\frac{w_{l,\left(i-1,j\right)}-w_{l,\left(i,j\right)}}{S}+D_{lT}\frac{T_{l,\left(i-1,j\right)}-T_{l,\left(i,j\right)}}{S}\right)\\ J_{l,\left(i+1,j\right)}=A_{sd}\rho_{l}\left(D_{lw}\frac{w_{l,\left(i+1,j\right)}-w_{l,\left(i,j\right)}}{S}+D_{lT}\frac{T_{l,\left(i+1,j\right)}-T_{l,\left(i,j\right)}}{S}\right)\\ J_{l,\left(i,j-1\right)}=A_{sd}\rho_{l}\left(D_{lw}\frac{w_{l,\left(i,j-1\right)}-w_{l,\left(i,j\right)}}{S}+D_{lT}\frac{T_{l,\left(i,j-1\right)}-T_{l,\left(i,j\right)}}{S}\right)\\ J_{l,\left(i,j+1\right)}=A_{sd}\rho_{l}\left(D_{lw}\frac{w_{l,\left(i,j+1\right)}-w_{l,\left(i,j\right)}}{S}+D_{lT}\frac{T_{l,\left(i,j+1\right)}-T_{l,\left(i,j\right)}}{S}\right)\\ J_{l,\left(i,j\right),up}=0 \end{array}$$

where $J_{l,\left(i-1,j\right)}$, $J_{l,\left(i+1,j\right)}$, $J_{l,\left(i,j-1\right)}$, and $J_{l,\left(i,j+1\right)}$ indicate water migration mass from unit (i−1,j), (i+1,j), (i,j−1), and (i,j+1) in unit time. $A_{sd}=SL$, $A_{u}=S^{2}$, where *S* is thermal well spacing and *L* is thermal well depth. $D_{lw}$ and $D_{lT}$ are called isothermal and thermal water diffusivities, respectively. $D_{lw}=K_{l}\frac{\partial \psi }{\partial w}$, $D_{lT}=K_{l}\frac{\partial \psi }{\partial T}$. The relationship of soil water potential with water content and temperature can be obtained by Gardner and De Vries [35]. $\psi =a{\left(\frac{w_{l}}{\varepsilon }\right)}^{-b}\exp\left(\gamma \cdot \left(T-273.15\right)\right)$, where *a*, *b* and γ are characteristic parameters of soils. ε indicates the porosity of soils.

(b) Vapor Flow Model

Similarly, for the mass conservation, the variable quantity of vapor in a certain unit block is equal to the sum between the amount of migration from surrounding units and the amount of internal evaporation. For a single unit block, the mass balance equation of vapor is:

(4) $$\begin{array}{c} V_{T,\left(i,j\right)}\frac{d\left(\rho_{v,\left(i,j\right)}w_{v,\left(i,j\right)}\right)}{d\tau }=E_{v,\left(i,j\right)}+\left(J_{v,\left(i-1,j\right)}+J_{v,\left(i+1,j\right)}+J_{v,\left(i,j-1\right)}+J_{v,\left(i,j+1\right)}+J_{v,\left(i,j\right),down}+J_{v,\left(i,j\right),up}\right)\\ +\left(J_{v,\left(i,j\right),e}+J_{v,\left(i,j\right),w}+J_{v,\left(i,j\right),s}+J_{v,\left(i,j\right),n}\right) \end{array}$$

where *ρ*_*v*_ is the density of vapor. $w_{v,\left(i,j\right)}$ indicates volume gaseous moisture content of the unit (i,j), $w_{v}=\frac{V_{v}}{V_{T}}$. $J_{v}$ is the mass of vapor migrated from adjacent units in unit time. $E_{v,\left(i,j\right)}=-E_{l,\left(i,j\right)}$.

Using $w_{l}+w_{v}=\varepsilon$, the left side of Equation (4) can be written as:

(5) $$\frac{d\left(\rho_{v}w_{v}\right)}{d\tau }=\frac{d\left(\rho_{v}\cdot \left(\varepsilon -w_{l}\right)\right)}{d\tau }=\left(\varepsilon -w_{l}\right)\frac{d\rho_{v}}{d\tau }-\rho_{v}\frac{dw_{l}}{d\tau }$$

Comparing Equations (4), (5), and (1), we have:

(6) $$\begin{array}{l} V_{T,\left(i,j\right)}\left(\varepsilon -w_{l,\left(i,j\right)}\right)\frac{d\rho_{v,\left(i,j\right)}}{d\tau }=\\ \frac{\rho_{v,\left(i,j\right)}}{\rho_{l}}(E_{l,\left(i,j\right)}+\left(J_{l,\left(i-1,j\right)}+J_{l,\left(i+1,j\right)}+J_{l,\left(i,j-1\right)}+J_{l,\left(i,j+1\right)}+J_{l,\left(i,j\right),down}+J_{l,\left(i,j\right),up}\right)\\ +\left(J_{l,\left(i,j\right),e}+J_{l,\left(i,j\right),w}+J_{l,\left(i,j\right),s}+J_{l,\left(i,j\right),n}\right))+E_{v,\left(i,j\right)}\\ +\left(J_{v,\left(i-1,j\right)}+J_{v,\left(i+1,j\right)}+J_{v,\left(i,j-1\right)}+J_{v,\left(i,j+1\right)}+J_{v,\left(i,j\right),down}+J_{v,\left(i,j\right),up}\right)\\ +\left(J_{v,\left(i,j\right),e}+J_{v,\left(i,j\right),w}+J_{v,\left(i,j\right),s}+J_{v,\left(i,j\right),n}\right) \end{array}$$

The mechanism of vapor migration in soil is mainly diffusion, which is a transitional diffusion of Fick and Kundsen diffusion. The vapor migration equation is [36]:

(7) $$j_{v}=-D_{e}\nabla \rho_{v}$$

where $j_{v}$ indicates vapor migration mass of unit area in unit time. $D_{e}$ is the vapor equivalent diffusivity. $\frac{1}{D_{e}}=\frac{1}{D_{atm}}+\frac{1}{D_{kn}}$. $D_{atm}$ and $D_{kn}$ indicate molecular diffusivity and Knudsen diffusivity, respectively.

In thermal remediation, there are vacuum wells to extract gases from soils. Extraction is treated as vapor migration on the upper surface of unit block. Therefore, there are two mechanisms of migration on the upper surface: the mass transfer of the upper surface and the extraction of vacuum wells.

According to continuum fluid dynamics theory, the flow velocity of gases, under the extraction of vacuum wells, can be described as the form of a modified Darcy’s law:

(8) $$j=-\frac{k_{rg}k}{\mu_{gv}}\left(\nabla p-\rho_{gv}g\right)$$

where *k* is intrinsic permeability of soils. $\mu_{gv}$ and p indicate viscosity and pressure of gas phase, respectively. $k_{rg}$ is relative permeability of gas phase, and it can be obtained through the Van Genuchten–Parker empirical formula [37]. $k_{rg}\left(S_{gv}\right)=S_{gv}^{1/2}{\left[1-{\left(1-S_{gv}\right)}^{1/m}\right]}^{2m}$, $S_{gv}$ is the saturation of gas phase, and *m* is an empirical parameter.

In addition, there is mass transfer on the surface of soils, which includes convective mass transfer and diffusion mass transfer. Assuming that the convective mass transfer coefficient is β, the vapor mass of convection is: $m_{vec}=\beta \left[p_{a}-p_{s}\right]$. According to Fick’s law, the mass of diffusion is $m_{D}=-D_{k}{\frac{\partial p_{v}}{\partial z}|}_{z=0}$ .

In summary, the amount of vapor migration on the soil surface is:

(9) $$J_{v,\left(i,j\right),up}=\frac{A_{ve}\rho_{v,\left(i,j\right)}k_{rg}k}{\mu_{gv}}\left(p_{ve}-p_{v,\left(i,j\right)}+\rho_{v,\left(i,j\right)}g\right)+\frac{A_{u}\left(p_{a}-p_{v,\left(i,j\right)}\right)}{\frac{L/2}{D_{k}}+\frac{1}{\beta }}$$

where $A_{ve}$ and $p_{ve}$ indicate the extraction area and pressure of the vacuum well. $p_{a}$ and $p_{v}$ are gas pressure in the atmosphere and soils.

For the lumped parameter method, the vapor fluxes of unit (i,j) with other adjacent units are:

(10) $$\{\begin{array}{l} J_{v,\left(i-1,j\right)}=A_{sd}D_{e}\left(\frac{\rho_{v,\left(i-1,j\right)}-\rho_{v,\left(i,j\right)}}{S}\right)\\ J_{v,\left(i+1,j\right)}=A_{sd}D_{e}\left(\frac{\rho_{v,\left(i+1,j\right)}-\rho_{v,\left(i,j\right)}}{S}\right)\\ J_{v,\left(i,j-1\right)}=A_{sd}D_{e}\left(\frac{\rho_{v,\left(i,j-1\right)}-\rho_{v,\left(i,j\right)}}{S}\right)\\ J_{v,\left(i,j+1\right)}=A_{sd}D_{e}\left(\frac{\rho_{v,\left(i,j+1\right)}-\rho_{v,\left(i,j\right)}}{S}\right)\\ J_{v,\left(i,j\right),down}=A_{u}D_{e}\left(\frac{\rho_{v,n}-\rho_{v,\left(i,j\right)}}{L/2}\right)\\ J_{v,\left(i,j\right),up}=\frac{A_{ve}\rho_{v,\left(i,j\right)}k_{rg}k}{\mu_{gv}}\left(p_{ve}-p_{v,\left(i,j\right)}+\rho_{v,\left(i,j\right)}g\right)+\frac{A_{u}\left(p_{a}-p_{v,\left(i,j\right)}\right)}{\frac{L/2}{D_{k}}+\frac{1}{\beta }}\\ J_{v,\left(i,j\right),e}=J_{v,\left(i,j\right),w}=J_{v,\left(i,j\right),s}=J_{v,\left(i,j\right),n}=A_{sd}D_{e}\left(\frac{\rho_{v,n}-\rho_{v,\left(i,j\right)}}{S/2}\right) \end{array}$$

where $J_{v,\left(i-1,j\right)}$, $J_{v,\left(i+1,j\right)}$, $J_{v,\left(i,j-1\right)}$, and $J_{v,\left(i,j+1\right)}$ indicate vapor migration mass from unit (i−1,j), (i+1,j), (i,j−1), and (i,j+1) in unit time. $J_{v,\left(i,j\right),e}$, $J_{v,\left(i,j\right),w}$, $J_{v,\left(i,j\right),s}$, $J_{v,\left(i,j\right),n}$, and $J_{v,\left(i,j\right),down}$ indicate vapor migration mass from unheated zones. $\rho_{v,n}$ is the vapor density in unheated zones.

(c) Heat Flow Model

**Figure 5:**
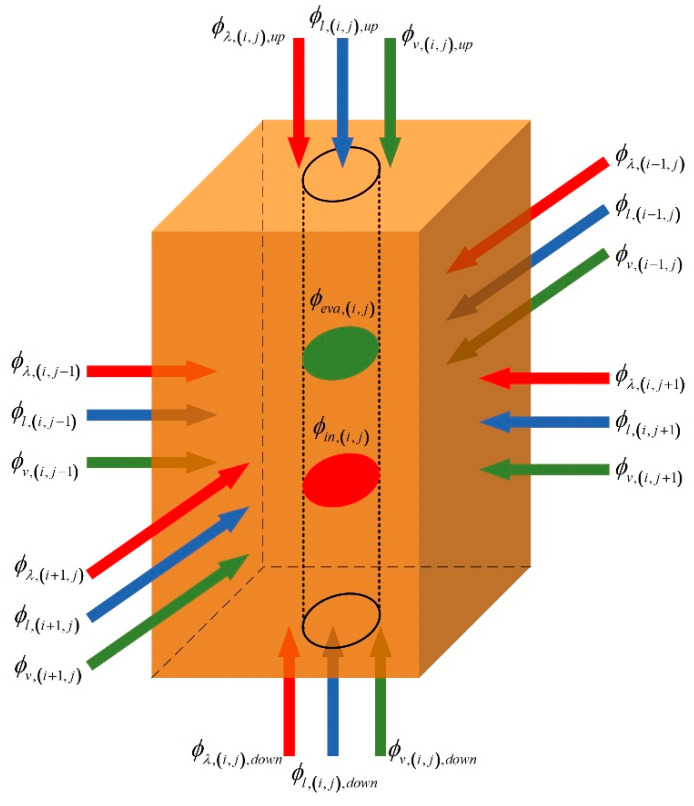
Heat flow of a unit block.

For the energy conservation, the enthalpy change in a certain unit block is equal to the sum of heat generated by thermal wells and the net heat flow through the unit. There are solid, liquid, and gas three phases in soils. Therefore, in addition to thermal conduction, the heat flow also includes heat flux caused by liquid migration and vapor migration. As Figure 5 shows, for a single unit block, the energy balance equation is:

(11) $$\begin{array}{l} M_{T,\left(i,j\right)}c_{T,\left(i,j\right)}\cdot \frac{dT_{s,\left(i,j\right)}}{d\tau }=\\ \phi_{in,\left(i,j\right)}+\left(\phi_{\lambda ,\left(i-1,j\right)}+\phi_{\lambda ,\left(i+1,j\right)}+\phi_{\lambda ,\left(i,j-1\right)}+\phi_{\lambda ,\left(i,j+1\right)}+\phi_{\lambda ,\left(i,j\right),down}+\phi_{\lambda ,\left(i,j\right),up}\right)\\ +\left(\phi_{l,\left(i-1,j\right)}+\phi_{l,\left(i+1,j\right)}+\phi_{l,\left(i,j-1\right)}+\phi_{l,\left(i,j+1\right)}+\phi_{l,\left(i,j\right),down}+\phi_{l,\left(i,j\right),up}\right)\\ +\left(\phi_{v,\left(i-1,j\right)}+\phi_{v,\left(i+1,j\right)}+\phi_{v,\left(i,j-1\right)}+\phi_{v,\left(i,j+1\right)}+\phi_{v,\left(i,j\right),down}+\phi_{v,\left(i,j\right),up}\right)+\phi_{eva,\left(i,j\right)}\\ +\left(\phi_{\lambda ,\left(i,j\right),e}+\phi_{\lambda ,\left(i,j\right),w}+\phi_{\lambda ,\left(i,j\right),s}+\phi_{\lambda ,\left(i,j\right),n}\right)+\left(\phi_{l,\left(i,j\right),e}+\phi_{l,\left(i,j\right),w}+\phi_{l,\left(i,j\right),s}+\phi_{l,\left(i,j\right),n}\right)\\ +\left(\phi_{v,\left(i,j\right),e}+\phi_{v,\left(i,j\right),w}+\phi_{v,\left(i,j\right),s}+\phi_{v,\left(i,j\right),n}\right) \end{array}$$

where $M_{T,\left(i,j\right)}$, $c_{T,\left(i,j\right)}$, and $T_{s,\left(i,j\right)}$ indicate total mass, mean specific heat, and temperature in the unit (i,j), separately. $M_{T,\left(i,j\right)}=V_{T,\left(i,j\right)}\left(\rho_{s}w_{s,\left(i,j\right)}+\rho_{l}w_{l,\left(i,j\right)}+\rho_{v}w_{v,\left(i,j\right)}\right)$.

*ϕ*_*in,(i,j)*_ indicates the heating power of thermal wells:

(12) $$\phi_{in,\left(i,j\right)}=P_{\left(i,j\right)}$$

where $P_{\left(i,j\right)}$ is the heat flowing into soils from the thermal well, which can be calculated by the model of thermal wells.

$\phi_{\lambda ,\left(i-1,j\right)}$, $\phi_{\lambda ,\left(i+1,j\right)}$, $\phi_{\lambda ,\left(i,j-1\right)}$, and $\phi_{\lambda ,\left(i,j+1\right)}$ indicate the heat flux by thermal conduction from adjacent units. $\phi_{\lambda ,\left(i,j\right),e}$, $\phi_{\lambda ,\left(i,j\right),w}$, $\phi_{\lambda ,\left(i,j\right),s}$, $\phi_{\lambda ,\left(i,j\right),n}$, and $\phi_{\lambda ,\left(i,j\right),down}$ indicate the heat flux by thermal conduction from unheated zones. They can be calculated on the basis of Fourier’s law.

In thermal remediation, the upper surface of soils will be covered with insulation, which is composed of concrete, insulation bricks, gravel layer, etc. Therefore, the thermal resistance of the heat exchange on the soil surface includes the thermal resistance inside soils, the thermal resistance of insulation, and the thermal resistance of heat convection on the upper surface, and solar radiation should also be considered. Thus:

(13) $$\phi_{\lambda ,\left(i,j\right),up}=\frac{A_{u}\left(T_{a}-T_{s,\left(i,j\right)}\right)}{R_{1}+R_{2}+R_{3}}+\alpha_{s}A_{u}\phi_{rad}$$

where $R_{1}=\frac{L/2}{\lambda_{s}}$, $R_{2}=\frac{\delta_{ins}}{\lambda_{ins}}$, and $R_{3}=\frac{1}{h_{a}}$. $\lambda_{ins}$ and $\delta_{ins}$ are thermal conductivity and thickness of the insulation. $h_{a}$ is the convective heat transfer coefficient. $\phi_{rad}$ indicates the energy radiated by sun on unit area, and $\alpha_{s}$ is the absorption rate of soils.

$\phi_{l}$ and $\phi_{v}$ indicate the heat flux through liquid migration and vapor migration, respectively.

(14) $$\{\begin{array}{l} \phi_{l}=H_{l}J_{l}\\ \phi_{v}=H_{v}J_{v} \end{array}$$

where $H_{l}$ and $H_{v}$ indicate the enthalpy of liquid water and vapor, $H_{l}=c_{l}\left(T-273.15\right)$, $H_{v}=c_{v}\left(T-273.15\right)+\gamma_{H_{2}O}$. $\gamma_{H_{2}O}$ is the latent heat of water. $J_{l}$ and $J_{v}$ can be calculated by Equations (3) and (10).

$\phi_{eva,\left(i,j\right)}$ indicates the energy absorbed by evaporation of water in soils. $\phi_{eva,\left(i,j\right)}=\gamma_{H_{2}O}E_{l,\left(i,j\right)}$.

(d) Three Phases of Soil Warming

**Figure 6:**
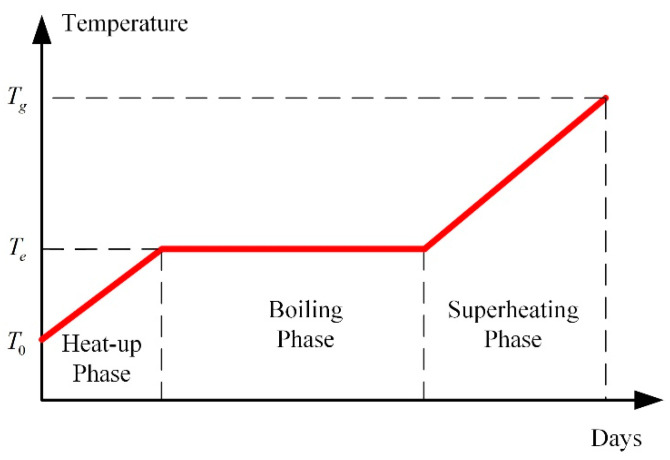
Three phases of soil warming.

During the heating process of thermal remediation, soil temperature can rise up to a very high value (generally around 350 °C [24]). A large number of experimental studies have shown that the temperature history of soils consists of three phases: heat-up phase, boiling phase, and superheating phase [38], as shown in Figure 6.

During the heat-up phase, the soil minerals and fluids (mainly water) are heated to the boiling point of water from the initial temperature. There are liquids and gases in the pores of soils. The evaporation of liquid water and liquid migration from unheated zones is negligible in this period.

During the boiling phase, the soil temperature stays at the boiling point until all the pore water has been boiled off. The energy generated by thermal wells is all converted into the latent heat required for evaporation of pore water. The duration of this phase depends on the amount of water to be boiled. When all the pore water has been vaporized, the dry soil can be superheated.

During the superheating phase, the dry soil is heated to the target temperature. The water infiltrated from surrounding units will be evaporated quickly because of the high temperature.

In short, the mechanisms of the three phases are not the same. In each phase, the liquid flow model, vapor flow model, and heat flow model should be slightly modified.

#### 2.2.2. Model of Thermal Well

**Figure 7:**
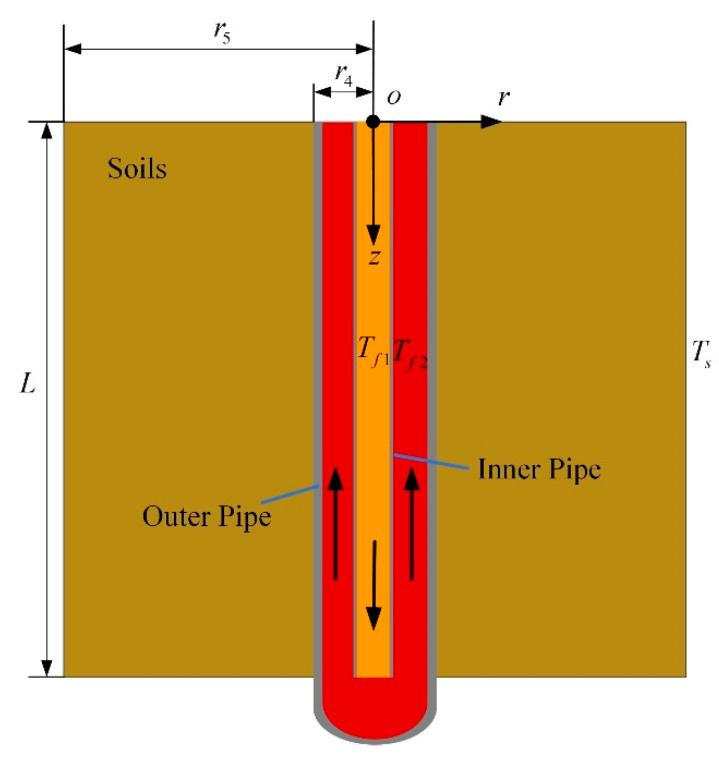
The structure diagram of thermal wells.

In order to obtain the heating power of thermal wells, the model of wells is established. Figure 7 shows the structure of thermal wells, which is composed of an inner pipe and an outer pipe, and an annular space is formed between inner pipe and outer pipe. High-temperature flue gases generated by combustors flow in through the inner pipe and then flow out through the outer pipe, thereby heating soils. In order to simplify the analytical process, it is assumed that the heat transfer in the thermal well is quasi-steady. Further, the heat transfer of the fluid in axial direction is ignored, and the heat transfer in the thermal well is considered to be one-dimensional in the radial direction. It is supposed that heat transfer of fluid in radial direction is converted into the temperature variation along the axial direction. Thus, the temperature relations of flue gases in the inner pipe and the annular space along axial direction are respectively established, and an analytical solution of the thermal well’s outlet temperature is obtained. The coordinates are set as shown in Figure 7. The origin of coordinates is located at the intersection of the axis of thermal wells and the ground surface.

Assumptions:

(1) Soil is homogeneous.
(2) The temperature and speed of the same section of fluid in thermal well are the same.
(3) In the model of thermal wells, the thermophysical parameters of soil are invariable, and moisture transfer is ignored.
(4) The energy loss at the junction of the inner pipe and the bottom of the outer pipe is not considered.
(5) The thermophysical parameters of flue gas do not change with temperature in thermal wells.

**Figure 8:**
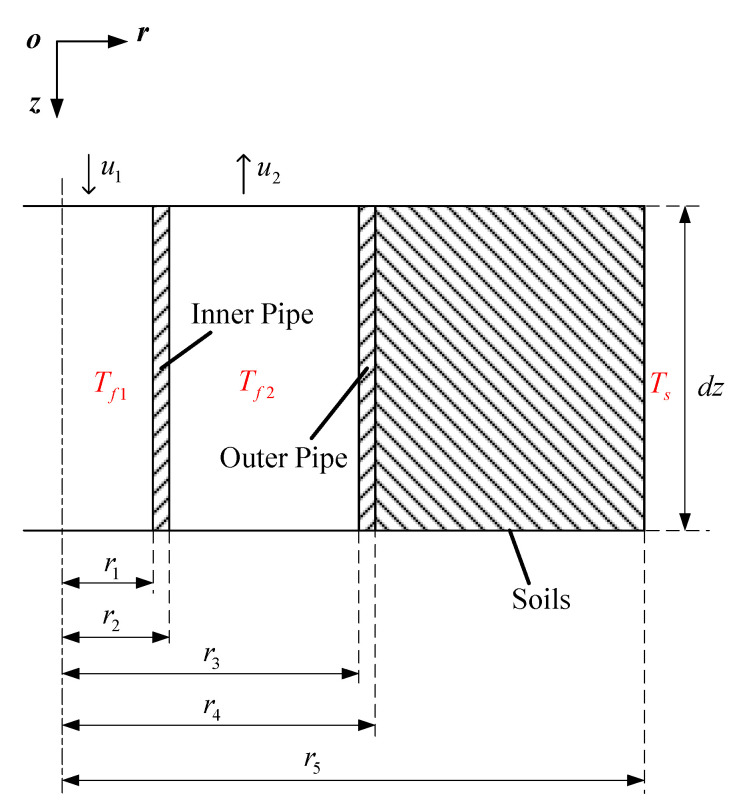
A microsegment of thermal wells.

A microsegment of thermal wells is shown in Figure 8. The heat transfer of the microsegment through the inner pipe is:

(15) $$dQ_{1}=\frac{2\pi dz(T_{f2}-T_{f1})}{\frac{1}{h_{1}r_{1}}+\frac{1}{\lambda_{1}}\ln\frac{r_{2}}{r_{1}}+\frac{1}{h_{2}r_{2}}}=\frac{2\pi dz(T_{f2}-T_{f1})}{R_{t1}}$$

where $R_{t1}=\frac{1}{h_{1}r_{1}}+\frac{1}{\lambda_{1}}\ln\frac{r_{2}}{r_{1}}+\frac{1}{h_{2}r_{2}}$. $h_{1}$ and $h_{2}$ are convective heat transfer coefficients of the inner pipe’s inner side and outer side, respectively. $r_{1}$ and $r_{2}$ are, respectively, the radius of the inner pipe’s inner side and outer side. $T_{f1}$ and $T_{f2}$ indicate the gas temperature in the inner pipe and annular space. $\lambda_{1}$ is the thermal conductivity of the inner pipe.

The enthalpy change of flue gases in the inner pipe is:

(16) $$dH_{1}=\rho_{g}c_{g}\frac{\partial T_{f1}}{\partial z}dz\cdot \pi r_{1}^{2}u_{1}$$

where $u_{1}$, $\rho_{g}$, and $c_{g}$ indicate the flow rate, density, and specific heat of flue gases in the inner pipe, separately.

The thermal balance: $dQ_{1}=dH_{1}$, then:

(17) $$\frac{2}{R_{t1}}\cdot (T_{f2}-T_{f1})-\rho_{g}c_{g}\cdot \frac{\partial T_{f1}}{\partial z}r_{1}^{2}u_{1}=0$$

A heat balance equation is also established for flue gases in the annular space. The heat exchange between flue gases in the annular space with soils is:

(18) $$dQ_{2}=\frac{2\pi dz(T_{s}-T_{f2})}{\frac{1}{h_{3}r_{3}}+\frac{1}{\lambda_{2}}\ln\frac{r_{4}}{r_{3}}+\frac{1}{\lambda_{3}}\ln\frac{r_{5}}{r_{4}}}=\frac{2\pi dz(T_{s}-T_{f2})}{R_{t2}}$$

where $R_{t2}=\frac{1}{h_{3}r_{3}}+\frac{1}{\lambda_{2}}\ln\frac{r_{4}}{r_{3}}+\frac{1}{\lambda_{3}}\ln\frac{r_{5}}{r_{4}}$. $h_{3}$ is the convective heat transfer coefficient of the outer pipe’s inner side. $r_{3}$ and $r_{4}$ are, respectively, the radius of the outer pipe’s inner side and outer side. $\lambda_{2}$ and $\lambda_{3}$ indicate the thermal conductivity of outer pipe and soils, respectively. $r_{5}$ is the effective radius of thermal wells. $r_{5}=\frac{S}{2}$, *S* indicates the thermal well spacing. $T_{s}$ is the soil temperature at $r_{5}$.

The enthalpy change of flue gases in annular space is:

(19) $$dH_{2}=-\rho_{g}c_{g}\pi (r_{3}^{2}-r_{2}^{2})u_{2}\frac{\partial T_{f2}}{\partial z}dz$$

where $u_{2}$ indicates the flue gases rate in annular space.

The thermal balance of flue gas in annular space is $-dQ_{1}+dQ_{2}=dH_{2}$, then:

(20) $$\frac{2}{R_{t2}}\cdot (T_{s}-T_{f2})-\frac{2}{R_{t1}}\cdot (T_{f2}-T_{f1})=-\rho_{g}c_{g}(r_{3}^{2}-r_{2}^{2})u_{2}\frac{\partial T_{f2}}{\partial z}$$

The flue gas density in the thermal well is assumed to be invariable, so the mass flow in the inner pipe is equal to that of annular space.

(21) $$r_{1}^{2}u_{1}=(r_{3}^{2}-r_{2}^{2})u_{2}$$

Boundary conditions are:

(22) $$\{\begin{array}{l} T_{f1}(0)=T_{fi}\\ T_{f2}(L)=T_{f1}(L) \end{array}$$

where $T_{fi}$ is the inlet temperature of thermal wells, which is assumed to be equal to the outlet temperature of combustors. *L* indicates the depth of inner pipe.

Solving Equations (17) and (20) simultaneously, we get:

(23) $$\{\begin{array}{l} T_{f1}(z)=(1+\zeta_{1})\chi_{1}e^{\psi_{1}z}+(1+\zeta_{2})\chi_{2}e^{\psi_{2}z}+T_{s}\\ T_{f2}(z)=\chi_{1}e^{\psi_{1}z}+\chi_{2}e^{\psi_{2}z}+T_{s} \end{array}$$

The outlet temperature of the thermal wells is:

(24) $$T_{fo}=T_{f2}(0)=\chi_{1}+\chi_{2}+T_{s}$$

Then, the heating power of the thermal well can be calculated as:

(25) $$P=G_{g}c_{g}\left(T_{fi}-T_{fo}\right)$$

where: $\{\begin{array}{l} R_{t1}=\frac{1}{h_{1}r_{1}}+\frac{1}{\lambda_{1}}\ln\frac{r_{2}}{r_{1}}+\frac{1}{h_{2}r_{2}}\\ R_{t2}=\frac{1}{h_{3}r_{3}}+\frac{1}{\lambda_{2}}\ln\frac{r_{4}}{r_{3}}+\frac{1}{\lambda_{3}}\ln\frac{r_{5}}{r_{4}} \end{array}$, $\{\begin{array}{l} \psi_{1}=\frac{1+\sqrt{1+\frac{4R_{t2}}{R_{t1}}}}{\rho_{g}c_{g}r_{1}^{2}u_{1}R_{t2}}\\ \psi_{2}=\frac{1-\sqrt{1+\frac{4R_{t2}}{R_{t1}}}}{\rho_{g}c_{g}r_{1}^{2}u_{1}R_{t2}} \end{array}$, $\{\begin{array}{l} \chi_{1}=\frac{(T_{s}-T_{fi})\zeta_{2}e^{\psi_{2}L}}{\zeta_{1}e^{\psi_{2}L}-\zeta_{2}e^{\psi_{1}L}}\\ \chi_{2}=\frac{(T_{s}-T_{fi})\zeta_{1}e^{\psi_{1}L}}{\zeta_{2}e^{\psi_{1}L}-\zeta_{1}e^{\psi_{2}L}} \end{array}$, $\{\begin{array}{l} \zeta_{1}=\frac{R_{t1}}{2R_{t2}}(1-\sqrt{1+\frac{4R_{t2}}{R_{t1}}})\\ \zeta_{2}=\frac{R_{t1}}{2R_{t2}}(1+\sqrt{1+\frac{4R_{t2}}{R_{t1}}}) \end{array}$.

#### 2.2.3. Model of Combustor

**Figure 9:**
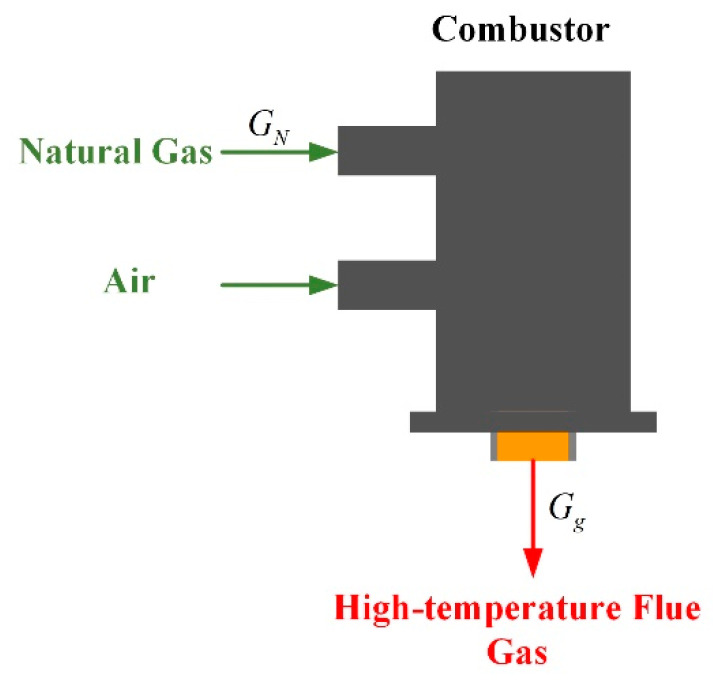
The schematic diagram of combustors.

In order to calculate the temperature, flow, and specific heat capacity of flue gases at the entrance of thermal wells, the model of a combustor was developed. The schematic diagram of the combustor is shown in Figure 9. Natural gas and air are burned in combustors to generate high-temperature flue gas. The outlet temperature and flow rate of flue gas can be obtained by establishing the mathematical model of chemical combustion in combustors. The natural gas is regarded as methane (CH4) in this study, and air is the combustion-supporting gas, which is composed of 78% nitrogen, 21% oxygen, and 1% other gases. Further, more air will be provided for the complete combustion of natural gas. In this paper, excess air ratio α is defined as the ratio of excess air to theoretical air. Then, the chemical equation can be obtained:

(26) $${\mathrm{CH}}_{4}+2(1+\alpha )O_{2}+7.429(1+\alpha )N_{2}\to {\mathrm{CO}}_{2}+2H_{2}O+7.429(1+\alpha )N_{2}+2\alpha O_{2}+Q$$

where *Q* indicates the heat generated by combustion.

The flue gas produced by methane combustion is a mixture of different gases. Its thermophysical parameters should be analyzed before the calculation of the flue gas’s temperature and flow. All gases are considered as ideal gases during the calculation.

The methane combustion is supposed to be complete. The proportion of each gas $w_{{\mathrm{CO}}_{2}}$, $w_{H_{2}O}$, $w_{N_{2}}$, and $w_{O_{2}}$ can be obtained based on the chemical equation.

The average specific heat of flue gas is:

(27) $$c_{g}=c_{{\mathrm{CO}}_{2}}\cdot w_{{\mathrm{CO}}_{2}}+c_{H_{2}O}\cdot w_{H_{2}O}+c_{N_{2}}\cdot w_{N_{2}}+c_{O_{2}}\cdot w_{O_{2}}$$

The specific heat capacity of gas is different at different temperatures. The molar specific heat of components in flue gases $c_{{\mathrm{CO}}_{2}}$, $c_{H_{2}O}$, $c_{N_{2}}$, and $c_{O_{2}}$ can be obtained according to the literature. Then:

(28) $$c_{g}=A+B\cdot (t_{1}+t_{2})$$

where $t_{1}$ and $t_{2}$ are initial centigrade temperature and final centigrade temperature of flue gases, respectively. $A=\frac{318.618+273.056\alpha }{10.429+9.429\alpha }$, $B=\frac{0.0404124+0.0254424\alpha }{10.429+9.429\alpha }$.

The density of gas at different temperatures is:

(29) $$\rho =\frac{M}{V}=\frac{pm_{mol}}{RT}$$

According to $\rho_{g}=\rho_{{\mathrm{CO}}_{2}}\cdot w_{{\mathrm{CO}}_{2}}+\rho_{H_{2}O}\cdot w_{H_{2}O}+\rho_{N_{2}}\cdot w_{N_{2}}+\rho_{O_{2}}\cdot w_{O_{2}}$, the density of flue gas is:

(30) $$\rho_{g}=\frac{32\times (18+17\alpha )}{16,629\times (10.429+9.429\alpha )}\cdot \frac{p}{T_{2}}$$

where $T_{2}$ indicates the outlet Kelvin temperature of combustors.

Input energy is equal to the energy of output according to conservation of energy, that is, the heat of methane combustion is equal to the energy required for the heating up of flue gases and the latent heat demanded by water vaporization. Moreover, there will be a certain loss of heat in combustion, so combustion efficiency ξ is introduced.

(31) $$G_{1}\xi q=G_{2}\left(c_{p,m}\cdot (t_{2}-t_{1})+w_{H_{2}O}\gamma_{H_{2}O}\right)$$

where $G_{1}$ indicates molar flow of natural gas. $G_{2}$ indicates molar flow of flue gases. $w_{H_{2}O}$ is the water content of flue gas. $\gamma_{H_{2}O}$ is the latent heat of water, $2.25\times 10^{6}\;J/\mathrm{kg}$. The low calorific value of methane combustion is 802.65 kJ/mol, so $q=8.0265\times 10^{5}\;J$.

After Equation (31) is solved, it can be obtained that:

$$t_{2}=\frac{-A+\sqrt{\frac{4B\xi qG_{1}}{G_{2}}+A^{2}-4Bw_{H_{2}O}\gamma_{H_{2}O}+4ABt_{1}+4B^{2}t_{1}^{2}}}{2B}$$

where $t_{1}$ is the initial temperature of gas, which is usually low, so it is supposed to be 0. Then, the outlet temperature of combustors is:

(32) $$t_{2}=\frac{-A+\sqrt{\frac{4B\xi qG_{1}}{G_{2}}+A^{2}-4Bw_{H_{2}O}\gamma_{H_{2}O}}}{2B}$$

According to Equation (26), the flue gas flow is:

(33) $$G_{g}=\frac{(9.429\alpha +10.429)\cdot T_{2}}{T_{1}}\cdot G_{N}$$

where $G_{g}$ is the volume flow of flue gases. $G_{N}$ indicates the natural gas volume flow. $T_{1}$ and $T_{2}$ are the Kelvin temperature of natural gas and flue gas, separately.

(34) $$T_{fi}=T_{2}$$

### 2.3. Fuzzy Coordination Control Strategy

**Figure 10:**
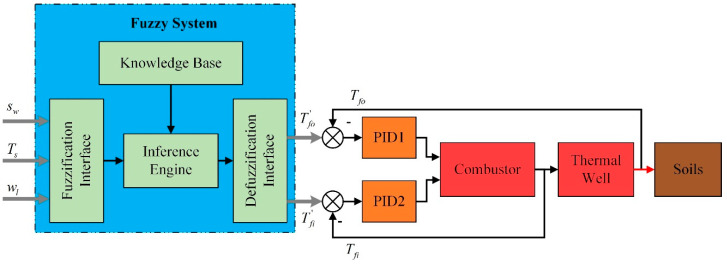
The graphical illustration of the fuzzy coordination control strategy.

The graphical illustration of the fuzzy coordination control strategy is presented in Figure 10. It accepts crisp mathematical values as input, and these crisp values are then converted into fuzzy sets by fuzzification interface [39]. The inference engine uses fuzzy rules presented in the knowledge base and generates output fuzzy sets. These fuzzy sets are then converted back to crisp values by the defuzzification interface. The fuzzy rules are defined by the system developer and vary from problem to problem [40].

**Figure 11:**
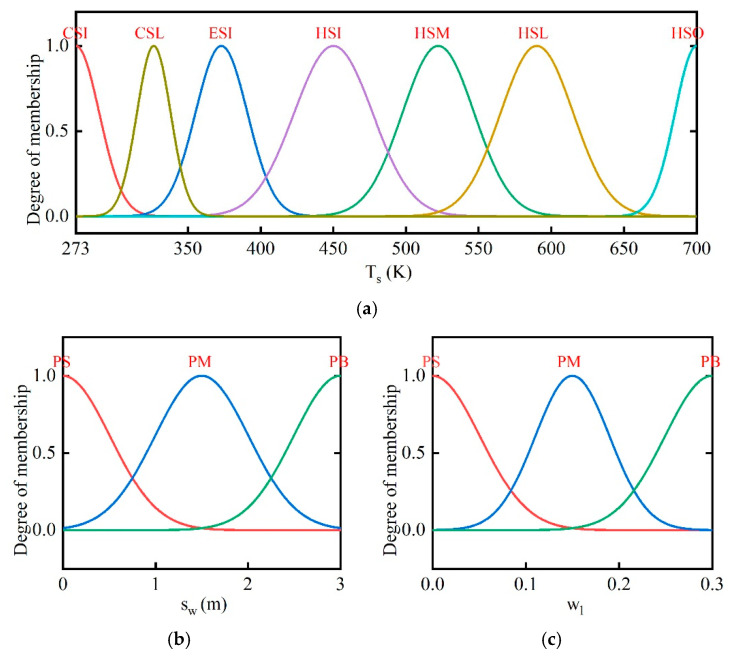
Membership functions of input variables. (**a**) Membership functions of soil temperature ; (**b**) membership functions of position $s_{w}$; (**c**) membership functions of water content $w_{l}$.

**Figure 12:**
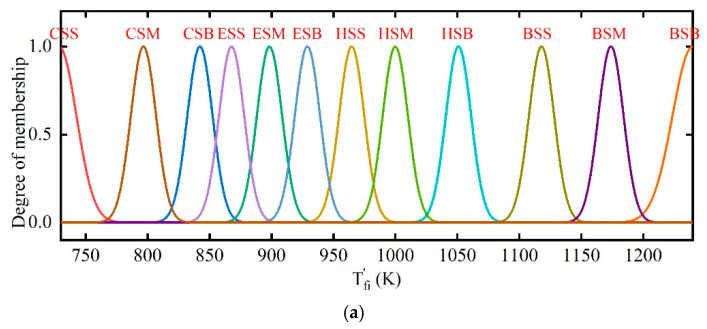

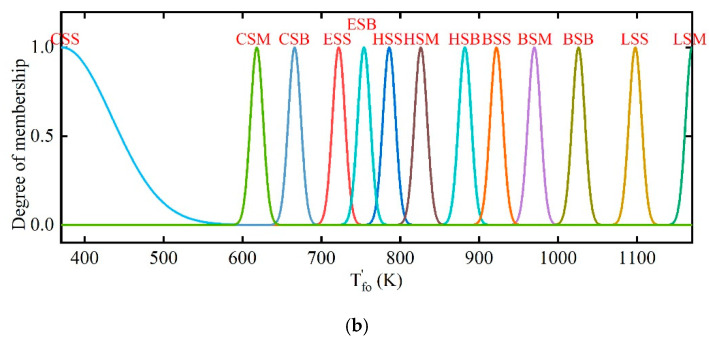
Membership functions of output variables. (**a**) Membership functions of desired outlet temperature in combustors $T_{fi}^{\prime }$; (**b**) membership functions of desired outlet temperature in thermal wells $T_{fo}^{\prime }$

The energy requirements of soils are variational at different locations. Thus, the position of thermal well *s_w_* is considered to reduce the impact of boundaries. $s_{w}$ indicates the sum of lateral and longitudinal distances from the thermal wells to the well on the border. Soil temperature $T_{s}$ and water content $w_{l}$ are considered to judge the warming phase of soils. The membership functions of input and output variables are given in Figure 11 and Figure 12. The Gaussian function has been used to ensure a smoother curve of output variables.

The output is determined using the fuzzy rules in the following form:

$$\mathrm{IF}\;s_{w}\;\mathrm{is}\;E_{i}\;\mathrm{and}\;T_{s}\;\mathrm{is}\;CE_{j}\;\mathrm{and}\;w_{l}\;\mathrm{is}\;CU_{k},$$

$$\mathrm{THEN}\;T_{fi}^{\prime }\mathrm{is}\;CT_{\left(i,j,k\right)}\;\mathrm{and}\;T_{fo}^{\prime }\;\mathrm{is}\;CP_{\left(i,j,k\right)}$$

where $E_{i}$, $CE_{j}$, $CU_{k}$, $CT_{\left(i,j,k\right)}$, and $CP_{\left(i,j,k\right)}$ are the fuzzy values of $s_{w}$, $T_{s}$, $w_{l}$, $T_{fi}^{\prime }$, and $T_{fo}^{\prime }$. There are 27 fuzzy rules in our fuzzy system. Because the water content changes during the boiling phase, the value of water content is considered only at this phase. Table 1 shows the complete list of fuzzy rules, which suggests the following:

(1) If the position of wells is closer to the boundary, the set values of combustors’ and wells’ outlet temperature should be higher.
(2) As the soil temperature increases and the water content decreases gradually, the desired outlet temperatures of combustors and thermal wells are set to be larger. The temperatures are set to be lower in the early stage and can reduce energy consumption.

## 3. Results and Discussion

The focus of this paper was to propose a coordination control strategy of multiple wells for the treatment zones, so as to reduce the energy consumption during the heating process without changing the duration. In addition to the fuzzy coordination control strategy, the energy consumptions of the field under the other three control strategies were also analyzed.

**Figure 13:**
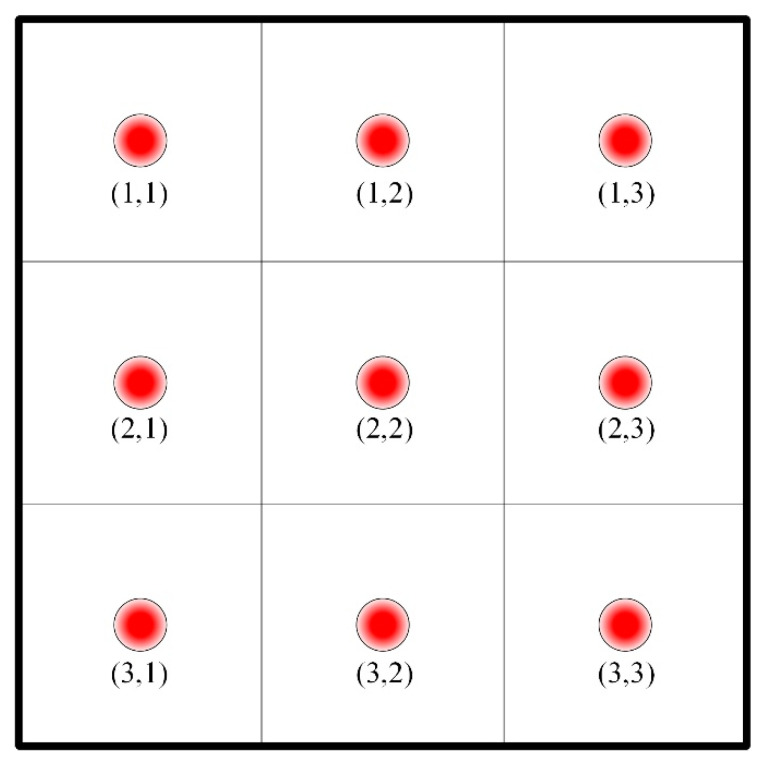
The pattern of nine thermal wells in numerical analysis.

The mathematical models presented above were built in MATLAB/Simulink, and the calculation results under different control strategies were obtained. Figure 13 represents the schematic diagram of the simulation model. Nine thermal wells are presented in a multiple rectangular array. The area is divided into different unit blocks based on the pattern of thermal wells. Within the model of each unit block, there are the model of heating devices and model of heating field. The model of heating field consists of three phases of temperature rise, and each phase contains three mathematical models: liquid flow model, vapor flow model, and heat flow model.

In this work, energy consumption is defined as the amount of natural gas consumed by the heating of soils. The energy consumption of a single thermal well can be calculated by:

(35) $$G_{ij}=\int_{0}^{\tau_{z}}G_{N,\left(i,j\right)}dt$$

where $G_{i,j}$ indicates the energy consumption of a single thermal well. $\tau_{z}$ is the heating time. $G_{N,\left(i,j\right)}$ is the natural gas flow at different time.

Then, the energy consumption of all thermal wells in the entire treatment zone is:

(36) $$G=\sum_{i=1}^{i_{\max}}\sum_{j=1}^{j_{\max}}G_{ij}$$

where $i_{\max}$ and $j_{\max}$ are the number of rows and columns of thermal wells’ pattern.

**Figure 14:**
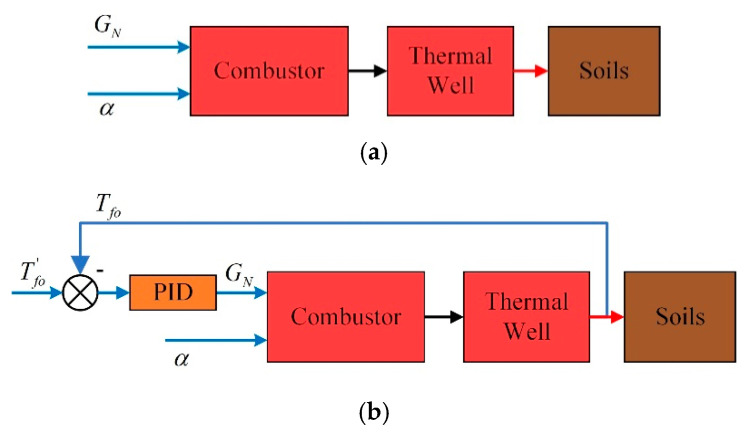

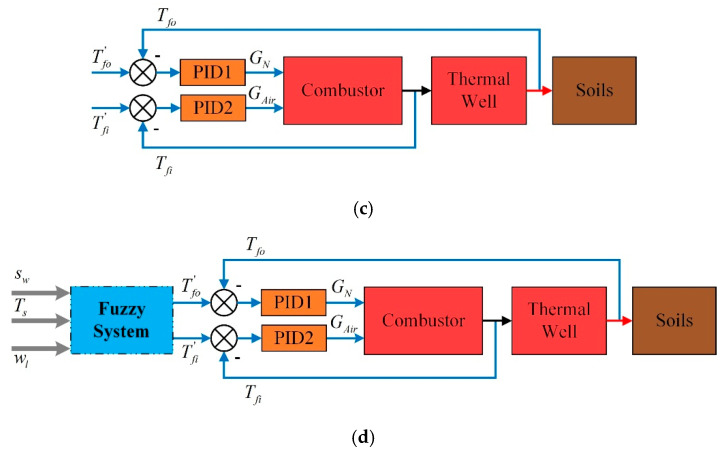
Control strategies of four cases. (**a**) Control strategy of case 1; (**b**) control strategy of case 2; (**c**) control strategy of case 3; and (**d**) control strategy of case 4.

In this paper, four cases were simulated in the MATLAB software. The structure diagrams of the four control strategies are shown in Figure 14. The differences are:

- Case 1: natural gas flow $G_{N}$ and excess air ratio α are constant;
- Case 2: the desired outlet temperature of thermal wells $T_{fo}^{\prime }$ and excess air ratio α are constant;
- Case 3: the desired outlet temperature of combustors $T_{fi}^{\prime }$ and thermal wells $T_{fo}^{\prime }$ are constant;
- Case 4: fuzzy coordination control strategy.

In addition to the control strategy, there are no differences in mathematical models and simulation parameters of four cases. The soil type in simulation is sandy soil; its properties are shown in Table 2.

Other simulation parameters are shown in Table 3. It can be seen that 101.25 m^3^ soils were treated during the process, which are about 194 tons.

### 3.1. Case 1: Constant Natural Gas Flow and Excess Air Ratio

**Figure 15:**
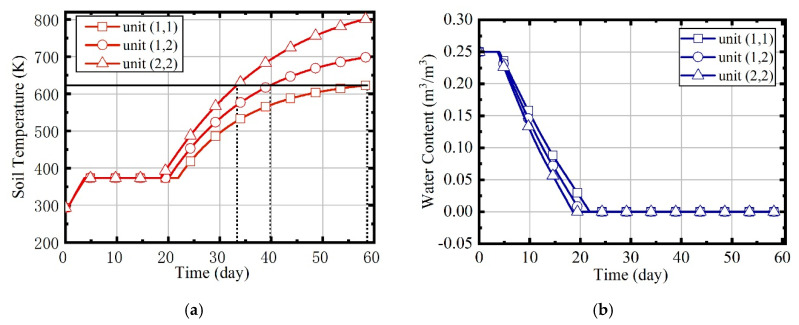
(**a**) The variation of soil temperatures in case 1; (**b**) the variation of water contents in case 1.

In case 1, natural gas flow and excess air ratio are constant during the heating process. The values are set to 2.05 × 10^−3^ m^3^/s and 1.5, respectively. Because the soil is assumed to be isotropic, parameters such as temperature and water content of the site are symmetrically distributed, so the units (1,1), (1,2), and (2,2) are mainly concerned. Figure 15 shows the variation of temperatures and water contents in units (1,1), (1,2), and (2,2). The temperature rise can be divided into three phases: heat-up phase, boiling phase, and superheating phase. The water content declines to 0 during the boiling phase.

The temperature rise curves of three units are quite different. Due to the influence of boundary heat leakage, unit (2,2) reaches the target temperature firstly, unit (1,2) is the second, and unit (1,1) is the last. The duration represents the heating time required to reach the target temperature. The durations of the three units are 58.62, 39.83, and 33.37 d, separately. The heating time of the site is supposed to be the largest duration of all units, and the temperature of unit (2,2) will continue to rise to 800 K, eventually.

**Figure 16:**
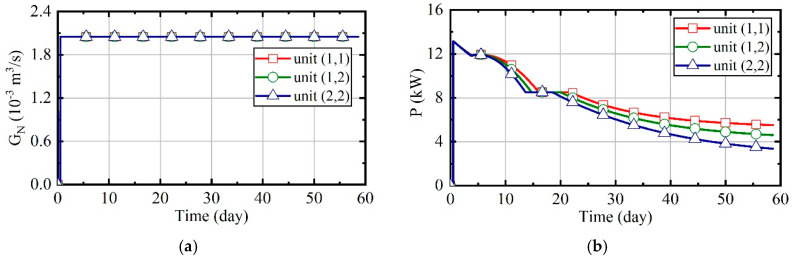

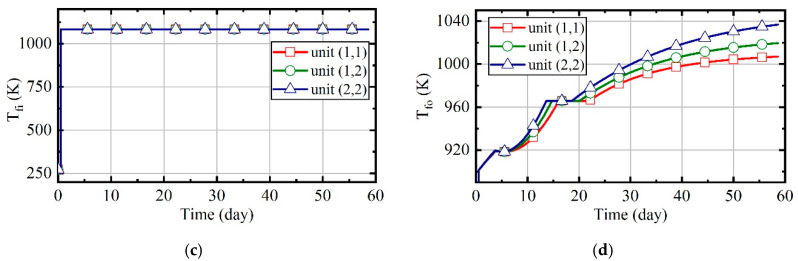
The analysis results of case 1. (**a**) Natural gas flow; (**b**) heating power of thermal wells; (**c**) outlet temperature of combustors; (**d**) outlet temperature of thermal wells.

Figure 16 illustrates the variation of natural gas flow, heating power, and outlet temperature of combustors and thermal wells over time for units (1,1), (1,2), and (2,2). In order to analyze the changes of parameters perfectly during the heating, there is no natural gas burning in the first half day of the simulation. In this case, the natural gas flow and excess air ratio of all units are constant, as Figure 16a shows. The outlet temperature of combustors is only related to the excess air ratio. Thus, it is invariable since the excess air ratio is constant, and it can be seen that it is about 1080 K from Figure 16c. Figure 16b represents that the heating power of thermal wells decreases over time. From Figure 16d, it can be observed that the outlet temperature of thermal wells increases over time. Soil thermal conductivity decreases as soil moisture content reduces. As the soil temperature increases and thermal conductivity decreases, the amount of heat absorbed by soils becomes less gradual. Therefore, when natural gas flow is constant, the outlet temperature of thermal wells will gradually increase. Meanwhile, because the soil temperature of unit (2, 2) is higher, there is a higher outlet temperature in its thermal well, and its heating power is lower.

**Figure 17:**
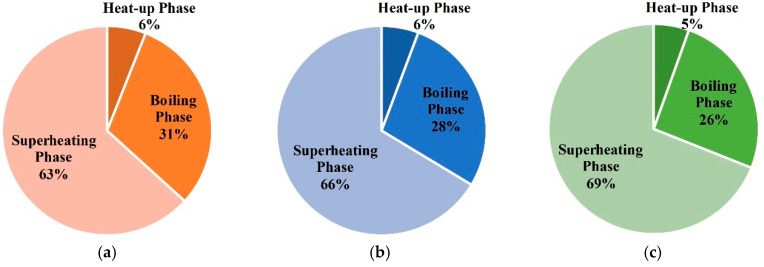
(**a**) The proportions of energy consumption during each phase for unit (1,1) in case 1; (**b**) the proportions of energy consumption during each phase for unit (1,2) in case 1; (**c**) the proportions of energy consumption during each phase for unit (2,2) in case 1.

The energy consumption of entire treatment zones is 92673 m^3^ in case 1. The proportions of energy consumption during each phase are shown in Figure 17. It can be seen that the superheating phase’s energy consumption is largest, and the heat-up phase’s energy consumption is very little. In this case, the natural gas flow remains unchanged. Thus, the proportion of energy consumption in different phases is mainly related to the duration of each phase.

### 3.2. Case 2: Constant Excess Air Ratio and Desired Outlet Temperature of Thermal Wells

In case 2, the excess air ratio and desired outlet temperature of thermal wells are constant during the heating process. They are set to 1.5 and 1000 K, respectively. The outlet temperature of thermal wells is taken as the controlled variable, and 1000 K is the set value. Further, the natural gas flow is adjusted by the deviation between the true value of the controlled variable and set value. This control method is most commonly used in engineering. 

**Figure 18:**
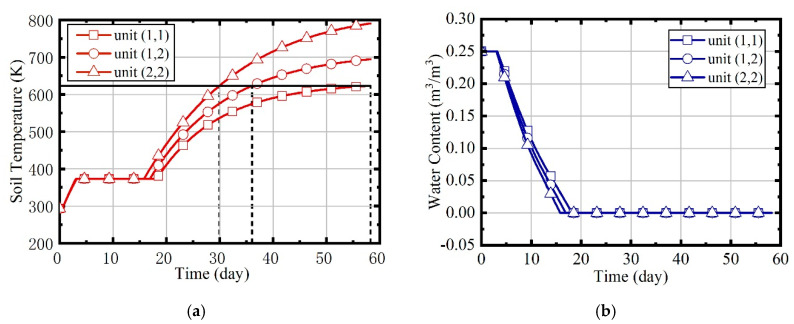
(**a**) The variation of soil temperatures in case 2; (**b**) the variation of water contents in case 2.

Figure 18 illustrates the variation of soil temperature and water content in case 2. The curves are very similar to those of case 1; the soil temperature rise can also be divided into three phases. The durations of units (1,1), (1,2), and (2,2) are 58.25, 36.05, and 29.94 d, separately. As soil temperature increases, the heating power decreases gradually. At the same time, the heat leakage also increases when temperature rises. Therefore, the warming trend of soils will gradually slow down in the superheating phase, which is especially obvious in unit (1,1) because of the effect of unheated zones.

**Figure 19:**
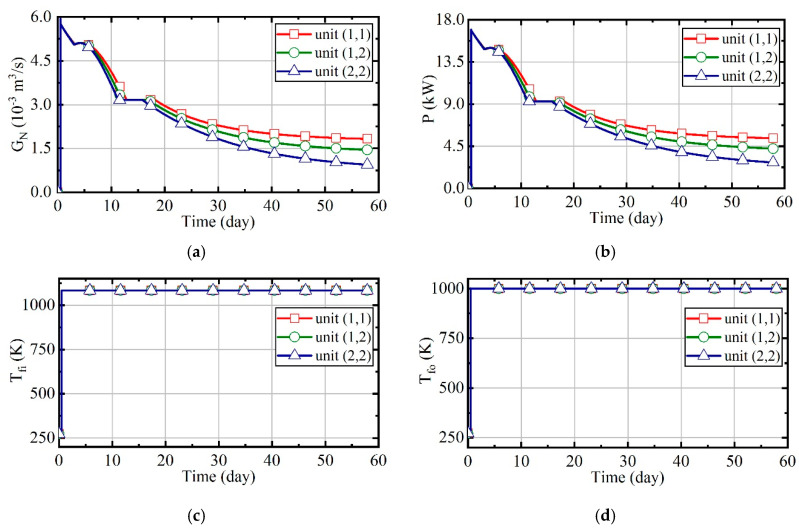
The analysis results of case 2. (**a**) Natural gas flow; (**b**) heating power of thermal wells; (**c**) outlet temperature of combustors; (**d**) outlet temperature of thermal wells.

Figure 19 represents the variation of natural gas flow, heating power, and outlet temperature of combustors and thermal wells over time for units (1,1), (1,2), and (2,2). In this case, the excess air ratio and desired outlet temperature of thermal wells are set to be unchanged, which can be seen in Figure 19c,d. As the soil temperature rises and thermal conductivity decreases, the amount of heat absorbed by soils becomes less gradual. Therefore, when outlet temperature of thermal wells is constant, the natural gas flow will gradually decrease, as Figure 19a shows. Meanwhile, because the soil temperature of unit (2,2) is highest, the natural gas flow is littlest in unit (2,2). From Figure 19b, it can be seen that the heating power decreases over time, and the value of unit (2,2) is the smallest.

**Figure 20:**
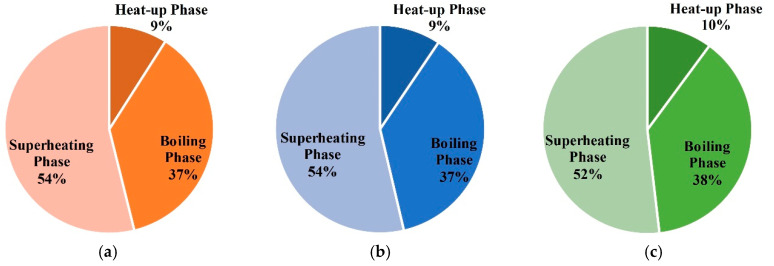
(**a**) The proportions of energy consumption during each phase for unit (1,1) in case 2; (**b**) the proportions of energy consumption during each phase for unit (1,2) in case 2; (**c**) the proportions of energy consumption during each phase for unit (2,2) in case 2.

The energy consumption of entire treatment zones is 119,250 m^3^ in case 2. The proportions of each phases are shown in Figure 20. In the process of thermal remediation, a mass of energy is consumed in the boiling phase and superheating phase. The energy consumption of the boiling phase can account for nearly 40%, so the moisture content has a great impact on the temperature rise of soils. Therefore, it will be necessary to control the water influx with temporary bulkheads, freeze walls, or de-watering in some sites.

### 3.3. Case 3: Constant Desired Outlet Temperature of Combustors and Thermal Wells

In case 3, the desired outlet temperatures of combustors and thermal wells are constant. The values are set to be 1100 and 1004 K, respectively. The flow of natural gas and air is adjusted according to the deviation of true value and set value of two temperatures, respectively.

**Figure 21:**
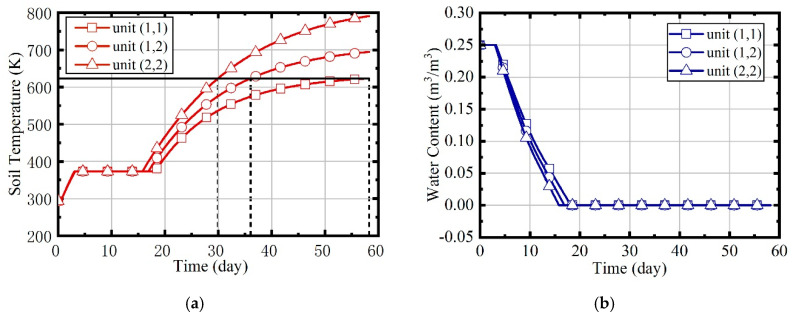
(**a**) The variation of soil temperatures in case 3; (**b**) the variation of water contents in case 3.

Figure 21 illustrates the variation of soil temperature and water content in case 3. The curves are the same as those of case 1 and case 2. The durations of units (1,1), (1,2), and (2,2) are 58.43, 36.14, and 29.99 d.

**Figure 22:**
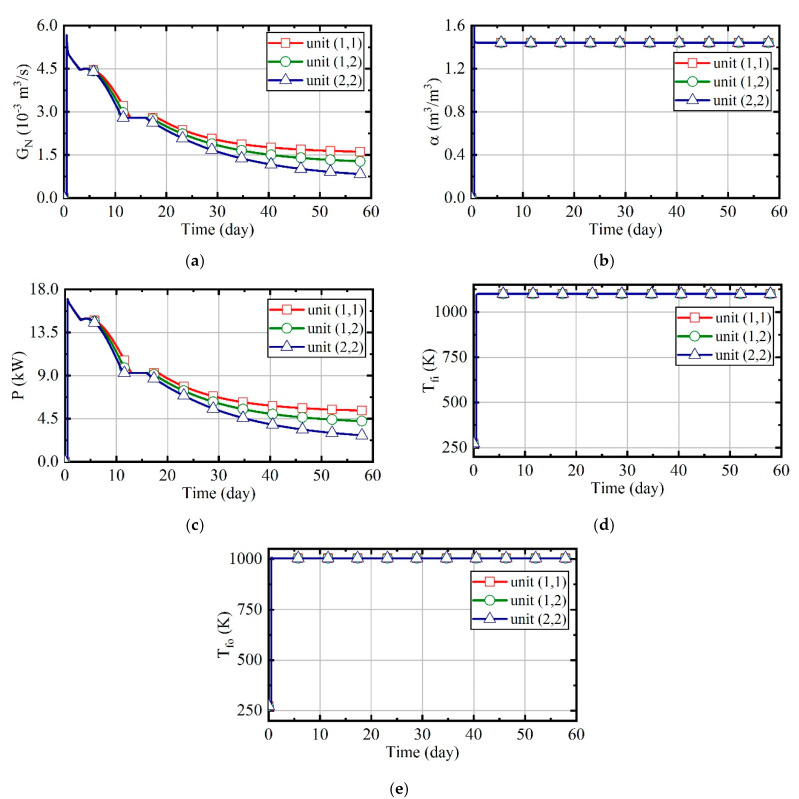
The analysis results of case 3. (**a**) Natural gas flow; (**b**) excess air ratio; (**c**) heating power of thermal wells; (**d**) outlet temperature of combustors; (**e**) outlet temperature of thermal wells.

Figure 22 represents the variation of natural gas flow, excess air ratio, heating power, and outlet temperature of combustors and thermal wells over time for units (1,1), (1,2), and (2,2). In this case, the desired outlet temperatures of combustors and thermal wells are set to be unchanged, which can be seen in Figure 22d,e. The outlet temperature of combustors is constant, so the excess air ratio is invariable over time, as Figure 22b shows. From Figure 22a,c, it can be seen that the natural gas flow and heating power gradually decreases over time, like that of case 2.

**Figure 23:**
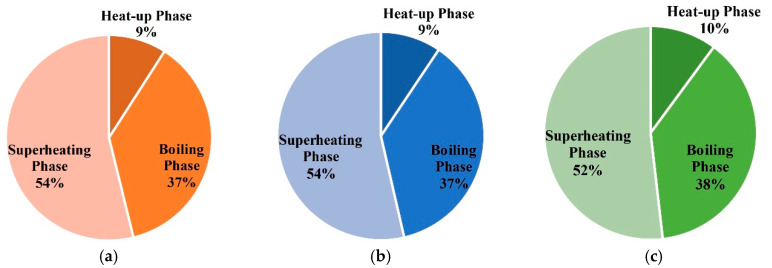
(**a**) The proportions of energy consumption during each phase for unit (1,1) in case 3; (**b**) the proportions of energy consumption during each phase for unit (1,2) in case 3; (**c**) the proportions of energy consumption during each phase for unit (2,2) in case 3.

The energy consumption of entire treatment zones is 105,730 m^3^ in case 3. The proportions of each phase are shown in Figure 23. Similarly, a mass of energy is consumed in the boiling phase and superheating phase.

### 3.4. Case 4: Fuzzy Coordination Control Strategy

**Figure 24:**
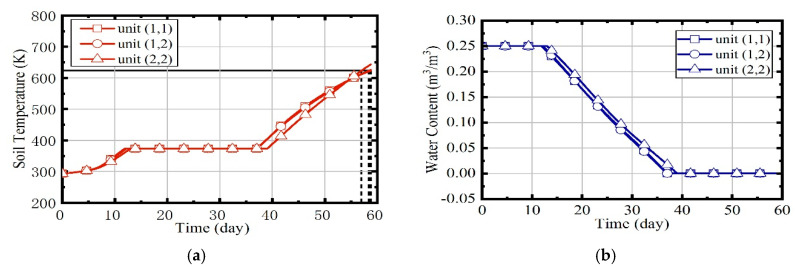
(**a**) The variation of soil temperatures in case 4; (**b**) the variation of water contents in case 4.

In case 4, the heating process of soils is controlled by the fuzzy coordination control strategy. The desired outlet temperature of combustors and thermal wells are adjusted by the fuzzy system. Figure 24 illustrates the variation of soil temperature and water content in case 4. It can be seen that the difference among three units’ temperature rise curves is very little after the adjustment of fuzzy system, and the three units reach the target temperature almost simultaneously. The durations of units (1,1), (1,2), and (2,2) are 58.75, 58.37, and 56.94 d. 

**Figure 25:**
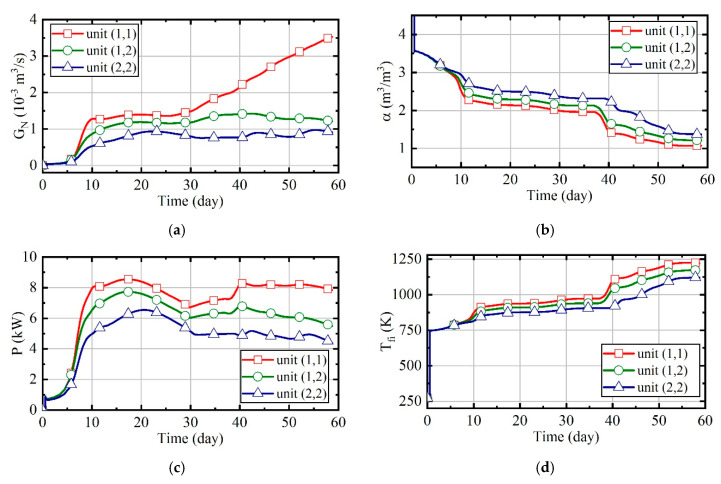

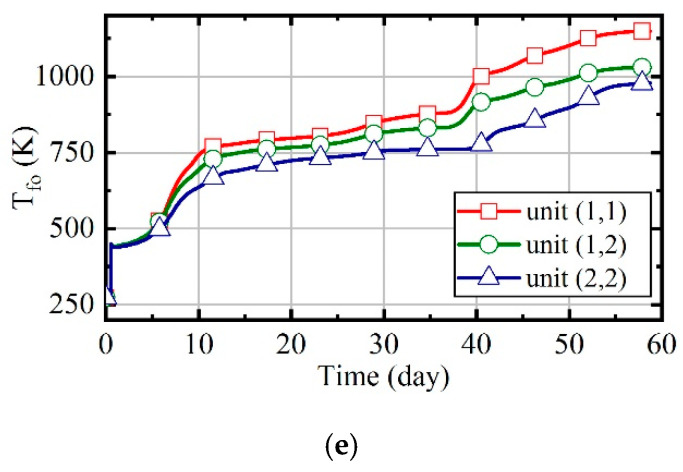
The analysis results of case 4. (**a**) Natural gas flow; (**b**) excess air ratio; (**c**) heating power of thermal wells; (**d**) outlet temperature of combustors; (**e**) outlet temperature of thermal wells.

Figure 25 represents the variation of natural gas flow, excess air ratio, heating power, and outlet temperature of combustors and thermal wells over time for units (1,1), (1,2), and (2,2). From Figure 25d,e, it can be observed that the outlet temperatures of combustors and thermal wells increase gradually with time after the adjustment of fuzzy system, and the closer the distance is to the boundary, the larger the values are. Therefore, the set values of unit (1,1) are largest among three units. The natural gas flow increases over time, and the excess air ratio reduces over time, as Figure 25a,b shows. From Figure 25c, it can be seen that the heating power keeps a small fluctuation. Since unit (1,1) has the largest amount of heat dissipation, its heating power is the highest.

**Figure 26:**
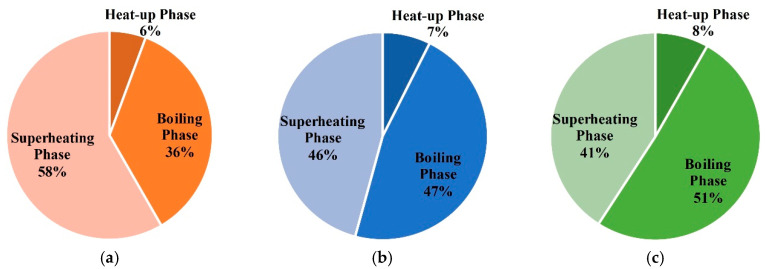
(**a**) The proportions of energy consumption during each phase for unit (1,1) in case 4; (**b**) the proportions of energy consumption during each phase for unit (1,2) in case 4; (**c**) the proportions of energy consumption during each phase for unit (2,2) in case 4.

The energy consumption of entire treatment zones is 61,217 m^3^ in case 4. The proportions of each phases are shown in Figure 26. Energy is mainly consumed in the boiling phase and superheating phase.

### 3.5. Comparison of Energy Consumption in Four Cases

**Figure 27:**
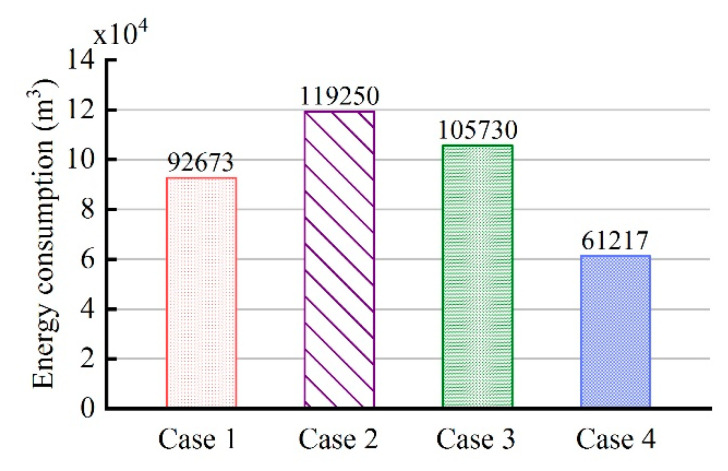
The total energy consumption of four cases.

After the simulation analysis of four cases, the energy consumption of different cases can be compared and analyzed to verify the superiority of the proposed control strategy for reducing energy consumption. Figure 27 illustrates the total energy consumption of the entire treatment zones in the four cases. It can be seen that the energy consumption of case 2 is the highest, 119,250 m^3^, and the energy consumption of case 4 is the lowest, 61,217 m^3^. Compared to that of case 1, the total energy consumption of cases 2 and 3 increased by 28.7% and 14.1%, respectively, while that of case 4 decreased by 33.9%. Meanwhile, the durations of the four cases are not much different, all being around 58 days. According to research, the control method of case 2 is commonly used by some companies, and compared to this method, the energy consumption of case 4 can be decreased by 48.7%. Therefore, the fuzzy coordination control strategy proposed in this paper can greatly reduce energy consumption without changing the total duration and achieve the energy-saving heating of soils.

**Figure 28:**
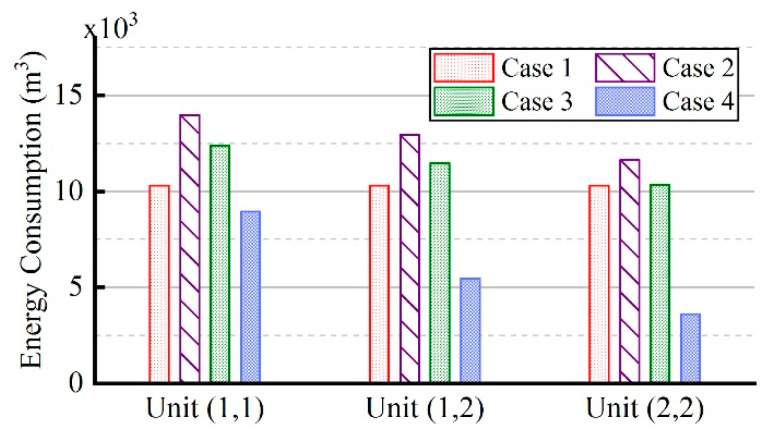
The energy consumption of each unit in four cases.

Figure 28 represents the energy consumption of each unit in four cases. Compared to that in case 1, the energy consumption of units (1,1), (1,2), and (2,2) in case 4 reduced by 13.2%, 46.9%, and 65.0%, separately. Further, among the three units, the degree of reduction in unit (2,2) is the highest. This is because in cases 1, 2, and 3, the difference in temperature rise of different units is large, and unit (2,2) will reach the target temperature as soon as possible. However, the entire site has not reached the target temperature, and the unit is still heated, so that its temperature will continue to rise, close to 800 K. Further, because of the large temperature difference among units, a considerable part of the energy will be transmitted to other units from unit (2,2), so the energy consumption is relatively high. In case 4, the temperature difference among different units is small, and the durations of different units are not much different, with less heat removed from unit (2,2). Therefore, the reduction proportion of unit (2,2) is the largest, and that of unit (1,1) is the littlest.

## 4. Conclusions

In order to reduce the energy consumption of in situ soil thermal remediation, this paper presented a thermohydraulic dynamics model and fuzzy coordination control strategy for a natural gas heating system. Multiple heating systems are controlled coordinately to heat 101.25 m^3^ soils in the treatment site. In addition to the fuzzy coordination control strategy, the other three traditional control strategies were also analyzed and compared. Several conclusions can be obtained:

(1) When the site is heated by indiscriminate energy, the soil at the boundary is warmed more slowly due to the larger heat dissipation;
(2) In the three phases of soil warming, the proportions of energy consumption in the boiling phase and superheating phase are the largest, and that of the heat-up phase is the smallest;
(3) Compared to the traditional control strategy I, that is, constant natural gas flow and excess air ratio, the fuzzy coordination control strategy can reduce energy consumption by 33.9%.
(4) Compared to the traditional control strategy II, that is, constant excess air ratio and desired outlet temperature of wells, the fuzzy coordination control strategy can reduce energy consumption by 48.7%.
(5) After the regulation of the fuzzy coordination control strategy, the soil heating rate is almost the same at different locations, and compared to soils in different locations, the energy consumption of soils located centrally was mostly reduced.

The results showed that the fuzzy coordination control strategy can greatly reduce energy consumption, thereby reducing the cost of in situ soil thermal remediation. This is conducive to the promotion and application of thermal remediation for the treatment of contaminated soils. Further, the models and methodology are valuable for different types of soils and different project requirements, which has a very important guiding significance for engineering application of thermal remediation.

## Figures and Tables

**Table 1:** Fuzzy decision rules.

$T_{s}$	$s_{w}$	$w_{l}$	$T_{fi}^{\prime }$	$T_{fo}^{\prime }$	$T_{s}$	$s_{w}$	$w_{l}$	$T_{fi}^{\prime }$	$T_{fo}^{\prime }$
CSI	PS	-	CSS	CSS	ESI	PB	PB	CSB	CSB
CSI	PM	-	CSS	CSS	HSI	PS	-	BSS	BSB
CSI	PB	-	CSS	CSS	HSI	PM	-	HSB	BSS
CSL	PS	-	CSM	ESS	HSI	PB	-	HSS	HSM
CSL	PM	-	CSM	CSB	HSM	PS	-	BSM	LSS
CSL	PB	-	CSM	CSM	HSM	PM	-	BSS	BSM
ESI	PS	PS	HSS	HSB	HSM	PB	-	HSB	HSB
ESI	PS	PM	ESB	HSS	HSL	PS	-	BSB	LSM
ESI	PS	PB	ESM	ESB	HSL	PM	-	BSM	BSB
ESI	PM	PS	ESB	HSM	HSL	PB	-	BSS	BSM
ESI	PM	PM	ESM	ESB	HSO	PS	-	CSB	ESS
ESI	PM	PB	ESS	ESS	HSO	PM	-	CSB	ESS
ESI	PB	PS	ESM	ESB	HSO	PB	-	CSB	ESS
ESI	PB	PM	ESS	ESS					

**Table 2:** Properties of soils.

Solid Density	Porosity	Saturated Hydraulic Conductivity	Specific Surface Area	Specific Heat of Dry Soils
2650 kg/m^3^	0.37	$6.3\times 10^{-6}\;m/s$	$100{\;m}^{2}{/m}^{3}$	1700 J/(kg·K)

**Table 3:** Parameters used in the calculation.

Parameters	Values	Parameters	Values
Initial soil temperature	20 °C	Moisture content	25%
Initial vapor density	0.748 kg/m^3^	Target temperature	350 °C
Well spacing *S*	1.5 m	Viscosity of gas phase in soils $\mu_{g}$	1.81 × 10^−5^ Pa·s
Well depth *L*	5 m	Latent heat of water $\gamma_{H_{2}O}$	2.25 × 10^6^ J/kg
Radius of inner pipe $r_{1}$	0.1 m	Radius of outer pipe $r_{3}$	0.15 m
Thickness of inner pipe	0.004 m	Thickness of outer pipe	0.0045 m
Pressure of vacuum wells $P_{ve}$	7000 Pa	Specific heat of liquid water $c_{l}$	4.2 × 10^3^ J/(kg·K)

## Nomenclature

**Symbol**			
*T*	Kelvin temperature (K)	α	Excess air ratio
*t*	Celsius temperature (℃)	*G*	Flow
*w*	content of material	$\gamma_{H_{2}O}$	Latent heat of water
*i*	Longitudinal arrangement number of the thermal well	*V*	Volume (m^3^)
*j*	Horizontal arrangement number of the thermal well	λ	Thermal conductivity (W/(m⋅K))
ρ	Density (kg/m^3^)	*u*	Flow rate of flue gases (m/s)
*h*	Convective heat transfer coefficient $\left(W/\left(m^{2}\cdot K\right)\right)$	*R*	Thermal resistance $\left(m^{2}\cdot K/W\right)$
τ	Time (s)	**Subscript**	
*E*	The mass of liquid evaporated in unit time (kg/s)	*s*	Soil particle
*J*	The mass of migration (kg/s)	*l*	Liquid
ψ	Soil water potential (J/kg)	*v*	Vapor
*A*	Area (m^2^)	*f*1	Flue gas in inner pipe
*D*	diffusivity (m/s)	*f*2	Flue gas in annular space
ε	Porosity of soils (m^3^/m^3^)	*g*	Flue gas
*p*	Pressure (Pa)	*fi*	Inlet of thermal wells
*L*	Thermal well depth (m)	*fo*	Outlet of thermal wells
*S*	Thermal well spacing (m)	*N*	Natural gas
*M*	Mass (kg)	*T*	Total
*c*	Specific heat capacity	*(i, j)*	The number of unit
ϕ	Heat flux (J/s)	*up*	Surface of soils
*P*	Heating power of thermal wells (kW)	*down*	Below the heating zone
*H*	Enthalpy (J/kg)	*sd*	Side of the unit block
*r*	Radius (m)	*lw*	Humidity gradient
*z*	Depth (m)	*lT*	Temperature gradient
*Q*	Heat (J)	*a*	Atmosphere
